# Supplementary material for: Quality Assessment of an Antimicrobial Resistance Surveillance System in a Province of Nepal
Source: Trop Med Infect Dis. 2021 Apr 23;6(2):60. doi: 10.3390/tropicalmed6020060 (PMC8167624; doi:10.3390/tropicalmed6020060)
Supplement: Supplementary file 1 [file tropicalmed-06-00060-s001.zip › tropicalmed-1149515-SI.pdf]

Tropical Medicine and Infectious Disease

Table S1. Priority specimens and pathogens for surveillance of AMR[1] according to GLASS[2] criteria for Nepal in 2019.

Specimen	Priority pathogens for surveillance
Blood	*Escherichia coli*<br>*Klebsiella pneumoniae*<br>*Acinetobacter baumannii*<br>*Staphylococcus aureus*<br>*Streptococcus pneumoniae*<br>*Salmonella* spp.[3]
Urine	*Escherichia coli*<br>*Klebsiella pneumoniae*
Stool	*Salmonella* spp.<br>*Shigella* spp.
Genital swabs	*Neisseria gonorrhoeae*

[1]AMR, antimicrobial resistance; [2] GLASS, Global Antimicrobial Resistance Surveillance System; [3]spp., species

Table S2. Pathogen–antibacterial combinations on which GLASS[1] gathers data.

Pathogen	Antibacterial agents that may be used for AST[2]
Escherichia coli	Ampicillin<br>Co-trimoxazole<br>Ciprofloxacin or levofloxacin<br>Ceftriaxone or cefotaxime and ceftazidime<br>Cefepime<br>Imipenem, meropenem, ertapenem, or doripenem<br>Colistin
Klebsiella pneumoniae	Co-trimoxazole<br>Ciprofloxacin or levofloxacin<br>Ceftriaxone or cefotaxime and ceftazidime,<br>Cefepime<br>Imipenem, meropenem, ertapenem, or doripenem<br>Colistin
Acinetobacter spp.[3]	Tigecycline or minocycline<br>Gentamicin and amikacin<br>Imipenem, meropenem, or doripenem<br>Colistin
Staphylococcus aureus	Cefoxitin<br>Oxacillin
Streptococcus pneumoniae	Penicillin G<br>Co-trimoxazole<br>Ceftriaxone or cefotaxime
Salmonella spp.	Ciprofloxacin or levofloxacin<br>Ceftriaxone or cefotaxime and ceftazidime<br>Imipenem, meropenem, ertapenem, or doripenem
Shigella spp.	Ciprofloxacin or levofloxacin<br>Ceftriaxone or cefotaxime and ceftazidime<br>Azithromycin
Neisseria gonorrhoeae	Cefixime

	Ceftriaxone<br>Azithromycin<br>Spectinomycin<br>Ciprofloxacin<br>Gentamicin

[1] GLASS, Global Antimicrobial Resistance Surveillance System; [2]AST: antibiotic susceptibility testing; [3]spp., species

Table S3. Basic infrastructure and specific requirements checklist for AMR[1] surveillance sites in Province 3 of Nepal (January to February 2020).

**Basic Infrastructure and Specific Requirements Checklist**			
	Name of the surveillance site lab:<br>Date of the supervising visit:<br>Name of supervisor:<br>Name of the surveillance site staff met:		
S.N.	Requirements	Response	Remarks
1	Number of microbiology staff		
2	Number of rooms dedicated to data entry		If none, skip next
3	Area of data entry room		In square feet<br>Adequate (>=150 sq.ft[2])[1]
4	Availability of computer for data entry		No/Yes/shared with other labs
5	Number of computers for data entry		
6	Availability of Internet service		If not, skip next field
7	Speed of Internet service		Less or more than 0.5 Mbps[3]
8	Availability of person for data entry		If yes, fill next field. If no, skip next field.
9	Qualification of data entry person		Name of degree
10	Training received on AMR Surveillance		If No, skip next field
11	When was AMR surveillance training received?		How many years ago? Mention the most recent
12	Training received on data entry and analysis		If no, skip next field
13	When was data entry training received?		How many years back?<br>Mention the most recent
14	Training received on WHONET[4]		If no, skip next field
15	When was WHONET training received?		How many years ago?<br>Mention the most recent
16	Agreement with NPHL[5]		Written/verbal /none
17	TOR[6] given to site		Written//none
18	Institutional restrictions on data sharing with NPHL		Written/verbal/none

[1]AMR, antimicrobial resistance; [2]sq.ft., square feet; [3]Mbps, megabytes per second; [4]WHONET, WHO freely downloadable software; [5]NPHL, National Public Health Laboratory; [6]TOR, terms of reference.

Reference:

1. National Public Health Laboratory NPHL. *Guideline on Health Laboratory Establishment and Operation*; Kathmandu, 2016.

Supplementary Document A1: Informed consent in local language taken from the participants during January to February 2020

अनुसन्धान सहभागिता को लागि मन्जुरीपत्र।

अनुसन्धान शिर्षक: "नेपाल को प्रदेश तीन मा एएमआर निगरानी डेटा कती राम्रो छ? एएमआर सेन्टिनल साइटहरूको पूर्णता र गुण प्रमाणीकरण जाँच।"

अनुसन्धानकर्ता को नाम: ज्योति आचार्य, राष्ट्रिय जनस्वास्थ्य प्रयोगशाला।

यस अध्ययनमा सहभागी हुँदा तपाइलाई कुनै हानी हुने वा असर पर्नेछैन। तपाईबाट संकलित सूचनाहरु अनुसन्धानसंग सम्बन्धित व्यक्तिहरुसंग मात्र गोप्य रहनेछ। तपाइको व्यक्तिगत जानकारी नामबाट नभई कोडबाट गरिनेछ। तपाईंलाई इच्छा नलागेमा कुनै पनि बेला सहभागिता परित्याग गर्नसक्नुहुनेछ। यस कारणले गर्दा तपाइले सम्बन्धित अस्पतालबाट प्राप्त गर्ने सेवा/सुबिधामा कुनै पनि असर पर्नेछैन।

सहभागीद्वारा घोषणा

तलहस्ताक्षरगरेर, म .... "नेपालको प्रदेश तीन मा एएमआर निगरानी डेटा कती राम्रो छ? एएमआर सेन्टिनलसाइटहरूको पूर्णता र गुणप्रमाणीकरण जाँच" शिर्षक को अनुसन्धान अध्ययनमा भाग लिन सहमत छु।

सहभागि/ अभिभावाककोनाम:...

हस्ताक्षर : ....

मिति: ....

Informed Consent

Declaration by participant

By signing below, I ………………………………………… agree to take part in a research study entitled "How good is AMR surveillance data in Province 3 of Nepal? A completeness and quality validation check of nine sentinel sites".

I declare that:

I have read this information and consent form and understand the contents.

I have had a chance to ask questions and all my questions have been adequately answered.

I understand that taking part in this study is voluntary and I have not been pressurized to take part.

I may choose to leave the study at any time and will not be penalized or prejudiced in any way.

Signed at (place): ....…........………… on (date) …………...………...

Signature of participant: Name:

.... Signature of witness

Declaration by researcher

I,Jyoti Acharya, declare that:

I explained the information in this document to ……………………………....

I encouraged him/her to ask questions and took adequate time to answer them.

I am satisfied that he/she adequately understands all aspects of the research, as discussed above

I did/did not use an interpreter (Sign the declaration below if an interpreter is used)

Signed at (place):________________________ on (date) ___________________________

_____________________ ___________________________

Signature of researcher Signature of witness

Declaration by interpreter (Not required for this study as all personnel are professionals who understand English)

I (name) ………………………………………….……… declare that:

I assisted the researcher (name) ……………………………………… to

explain the information in this document to (name of participant)

……………..…………………………….. using the language medium of .... (state which)

I conveyed a factually correct version of what was related to me.

I encouraged him/her to ask questions and took adequate time to answer them.

I am satisfied that the participant fully understands the content of this informed consent document and has had all his/her question satisfactorily answered.

Signed at (place)___________________________on (date)_____________________

_____________________ _________________

Signature of interpreter Signature of witness
